# The Effect of the Reducing Sugars in the Synthesis of Visible-Light-Active Copper(I) Oxide Photocatalyst

**DOI:** 10.3390/molecules26041149

**Published:** 2021-02-21

**Authors:** Szilvia Fodor, Lucian Baia, Kornélia Baán, Gábor Kovács, Zsolt Pap, Klara Hernadi

**Affiliations:** 1 Department of Applied and Environmental Chemistry, University of Szeged, Rerrich tér 1, 6720 Szeged, Hungary; fod_szilvia@chem.u-szeged.hu (S.F.); kornelia.baan@chem.u-szeged.hu (K.B.); 2 Nanostructured Materials and Bio-Nano-Interfaces Centre, Institute for Interdisciplinary Research on Bio-Nano-Sciences, Treboniu Laurian 42, 400271 Cluj-Napoca, Romania; lucian.baia@phys.ubbcluj.ro (L.B.); gkovacs@chem.ubbcluj.ro (G.K.); 3 Faculty of Physics, Babeș–Bolyai University, M. Kogălniceanu 1, 400084 Cluj-Napoca, Romania; 4 Institute of Environmental Science and Technology Tisza Lajos krt. 103, 6720 Szeged, Hungary; 5 Institute of Research-Development-Innovation in Applied Natural Sciences, Babes-Bolyai University, Fântânele 30, 400294 Cluj-Napoca, Romania; 6 Institute of Physical Metallurgy, Metal Forming and Nanotechnology, University of Miskolc, 3515 Miskolc-Egyetemváros, Hungary

**Keywords:** reducing sugars, copper(I) oxide, visible light activity, photocatalysts, shape tailoring

## Abstract

In the present work, shape tailored Cu_2_O microparticles were synthesized by changing the nature of the reducing agent and studied subsequently. d-(+)-glucose, d-(+)-fructose, d-(+)xylose, d-(+)-galactose, and d-(+)-arabinose were chosen as reducing agents due to their different reducing abilities. The morpho-structural characteristics were studied by X-ray diffraction (XRD), scanning electron microscopy (SEM), and diffuse reflectance spectroscopy (DRS), while their photocatalytic activity was evaluated by methyl orange degradation under visible light (120 min). The results show that the number of carbon atoms in the sugars affect the morphology and particle size (from 250 nm to 1.2 µm), and differences in their degree of crystallinity and photocatalytic activity were also found. The highest activity was observed when glucose was used as the reducing agent.

## 1. Introduction

One of the most promising approaches to convert solar energy into chemical energy is heterogenous photocatalysis [1,2]. The photocatalytic degradation of organic pollutants in wastewater can be achieved by using different semiconductors irradiated with ultraviolet or visible light [3].

Due to the practical usage of natural solar energy for wastewater treatment, the development of visible-light-active semiconductors is preferable. To this end, different types of photocatalysts were created, of which the most researched are perhaps the SnS_2_ [4], ZnS [5], BiO_X_ (X = Cl, Br, I) [6], and MWO_4_ (M^2+^ = Co, Cu, Pb, Cd, Mn, and Zn) [7]. An example of promising results using vis-light-active photocatalysts were presented by Ning et al. [8]. Many parameters can affect the photocatalytic activity, this is the reason why several scientific publications focus on this feature.

In solution-phase synthesis of Cu_2_O crystals, a typical route is the simple reduction of copper (II) salts [9,10]. By modifying the experimental parameters (e.g., temperature, stabilizing agent, or reducing agent) investigated so far, significant structural and morphological differences were induced and thus, the photocatalytic activity of the semiconductors could be increased [11]. The morphology [12], optical properties [13,14,15], crystal structure [16,17], and applicability [18,19] of semiconductors can also be controlled by varying the previously listed synthesis parameters.

One of the parameters just mentioned can be the temperature [20] or the stabilizing agent [9,10]. As mentioned above, different precursor materials can be used, which can lead to changes in the properties of the product as well [9].

The amount and the type of the reducing agents may also have an important influence, similar to the effect of compounds used in noble metal nanoparticles’ synthesis [21,22]. The most commonly used reducing agents in the synthesis of noble metal nanoparticles are hydrazine and borohydride (strong reducing agents) or sometimes hydrogen gas for slower reduction, which result in different particle shapes [23,24,25].

Typical reducing agents for the synthesis of Cu_2_O are ascorbic acid [26,27,28] and glucose [29,30,31,32]. However, a systematic study concerning the influence of different reducing agents is still missing.

Since photocatalysis can be considered/viewed as a green chemical water purification process regarding some aspects, it is important for these semiconductors to be produced in the most environmentally friendly manner possible. As examples, in their environmentally friendly synthesis, glucose and other aldose and ketose sugars were demonstrated as promising candidates for reducing agents [33].

For this reason, in this study, the production of Cu_2_O semiconductors was accomplished by chemical reduction with sugars having different carbon chain length, which has also been demonstrated by Kumbhar, P et al. [34]. The impact of these compounds on the characteristics of the semiconductors and their photocatalytic behavior was studied.

## 2. Results

### 2.1. Investigation of Structural Properties of Cu_2_O Samples—Dependence on Reducing Agent

**Figure 1:**
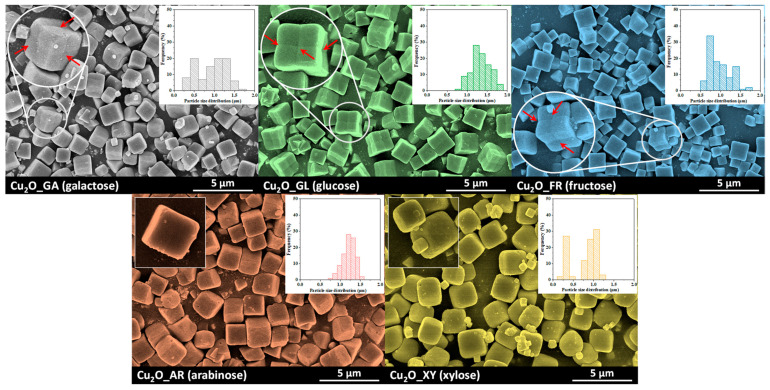
Scanning electron microscopy (SEM) micrographs of Cu_2_O microcubes obtained from CuCl_2_ precursor; the effect of the reducing sugars (top row: sugars containing six carbon atoms; bottom row: sugars containing five carbon atoms) can be observed in the size distribution and the morphology; the inset figure on the upper left for each sample contains the SEM micrograph of a single particle, while the particle size distribution is presented in the upper right corner of each micrograph.

Scanning electron microscopy was used to study the morphology of the Cu_2_O microparticles. From the SEM micrographs shown in Figure 1, a microcubic Cu_2_O morphology can be observed, which could be related to the ethylenediaminetetraacetic acid (EDTA) complexing agent used in the synthesis [10,30]. The particle size distributions are presented in the inserted histograms of the samples.

From the histograms, the predominant size was ~1.2 µm in most cases. The exceptions were sample Cu_2_O_GA, where the size distribution of the particles was wide (0.5–1.5 µm), and sample Cu_2_O_XY, where smaller particles with a size of 250 nm and larger particles with a size of 1.2 µm could be simultaneously observed.

Furthermore, the SEM micrographs show another interesting morphological feature: in case of a reducing sugar of six carbon atoms (Cu_2_O_GA, Cu_2_O_GL, Cu_2_O_FR)—additional edges appeared on the microcubes’ faces; this phenomenon was not observed in the samples where the reducing sugar contained five carbon atoms.

The degree of the delimiting planes which formed the new edge was calculated using ImageJ. The highest value was registered for fructose (16°), followed by glucose (9°), while the smallest angle was in the case of galactose (4°). After analyzing the morphology of the particles, the crystal structure of the particles was determined by X-ray diffractometry (XRD).

**Figure 2:**
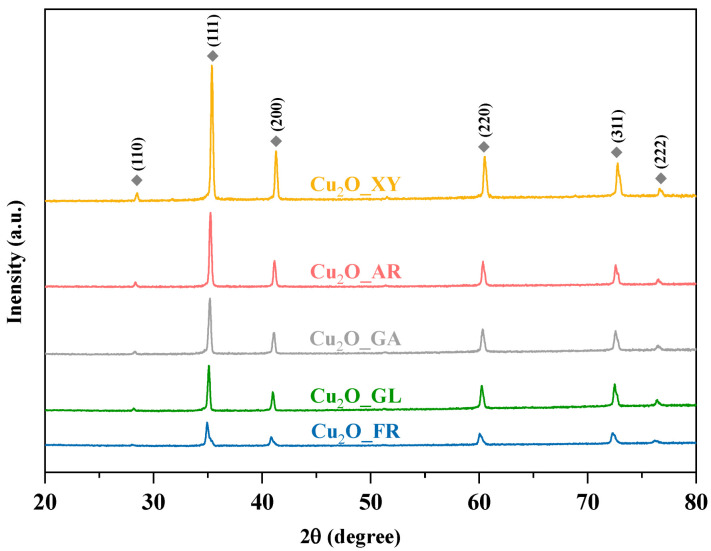
XRD patterns of the Cu_2_O samples—all the characteristic diffractions of Cu_2_O were marked with a grey rectangle.

The XRD patterns of the microcubes are shown in Figure 2. All the diffractions of the samples observed at 2θ° values of 29.40°, 36.33°, 42.16°, 61.47°, 73.35°, and 77.41° can be attributed to the characteristic diffractions of cubic Cu_2_O (JCPDS file no. 05–0667), which correspond to the (110), (111), (200), (220), (311), and (222) crystallographic planes, respectively. From the XRD patterns, no other characteristic signals of CuO or Cu can be detected, indicating that pure Cu_2_O was obtained.

As the signal/noise ratio increases, the degree of crystallinity decreases in the diffractograms (from top to bottom, Figure 2).

**Figure 3:**
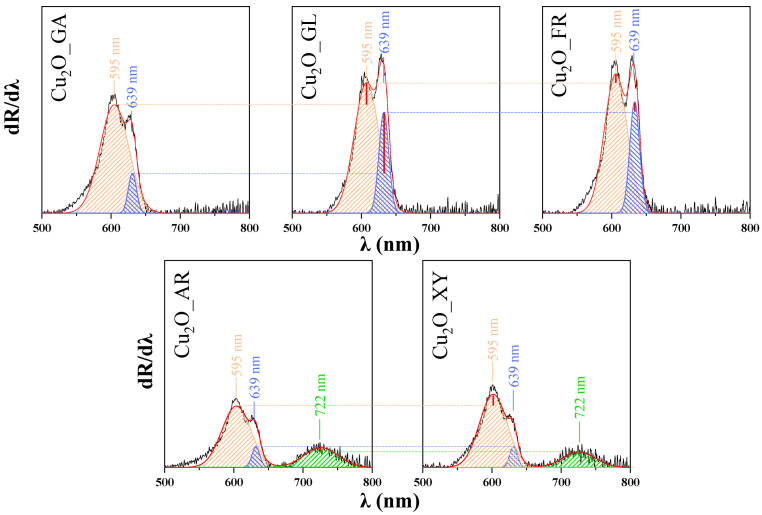
The deconvolution of the first order-derivative diffuse reflectance spectroscopy (DRS) spectra of the synthesized Cu_2_O samples (500–800 nm).

To investigate the optical properties of the Cu_2_O materials, their diffuse reflectance spectroscopy (DRS) spectra were recorded, then the first derivatives of the spectra were plotted (Figure 3) and deconvoluted. The deconvolution revealed the appearance of three different peaks located at 595 nm (2.08 eV), 639 nm (1.94 eV) and 722 nm (1.71 eV). The peak corresponding to the band gap value of Cu_2_O was observed at 1.94 eV (at 639 nm) but several other possible electron transitions could be assigned based on the literature [35].

As detailed in the discussion of SEM micrographs, influence of the reducing sugars’ carbon chain length (five vs. carbon six carbon atoms) was observed. In the DRS spectra of Cu_2_O_XY and Cu_2_O_AR, a new band appeared at 722 nm assigned to the localized surface plasmon resonance (LSPR) [36] of Cu_2_O. The broad appearance of this LSPR band is in agreement with the literature [37,38,39,40]. Localized surface plasmon resonance can be observed at increased concentration of free carriers (holes) in the material due to the introduction of copper vacancies, resulting in the increase of hole concentration as well [41,42].

The LSPR is related to and dependent on the degree of crystallinity [35]. This is in agreement with the observations reported here, as the specific LSPR band was observed only in the case of samples with the highest crystallinity (Cu_2_O_XY and Cu_2_O_AR).

The peak located at 595 nm can be explained by the appearance of the excitonic bandgap, which can form when the semiconductor absorbs a photon of higher energy than its own band gap [40]. The exciton band for Cu_2_O is located around 2.1 eV [40,41].

### 2.2. Investigation of the Effect of Reducing Sugars on the Photocatalytic Activity of Cu_2_O Samples

**Figure 4:**
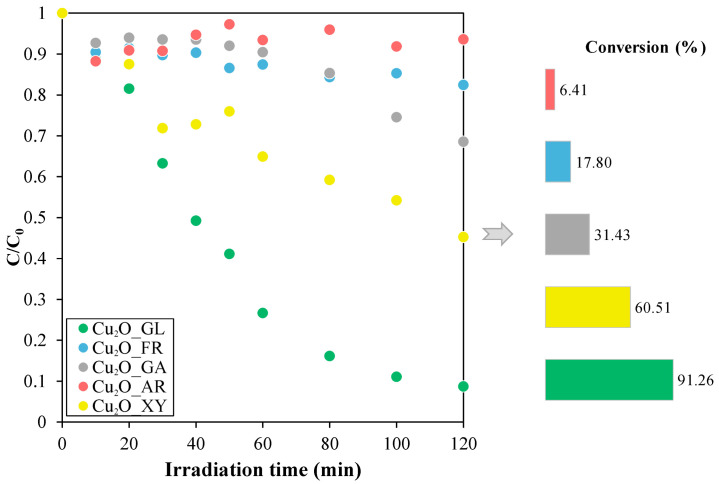
Degradation curves of methyl orange during the photocatalytic degradation tests under visible light irradiation.

After the physico-chemical characterization of the samples, their photocatalytic performance was determined using visible light irradiation, using methyl orange as model pollutant. As it was mentioned in the introduction, photocatalytic oxidation is an important purification process that involves a light-activated catalyst which reacts with adsorbed organic pollutants to oxidize them. Essentially, these molecules undergo a chemical reaction that transforms them into harmless substances. As shown in Figure 4, the efficiency of the methyl orange (MO) photodegradation by the different Cu_2_O samples showed promising results.

Significant differences were observed among the photocatalytic activities of the samples. In case of the arabinose-reduced sample (Cu_2_O_AR), the photocatalytic activity was negligible with a conversion of only 6.4%. The highest activity, 91.2%, was observed with the Cu_2_O reduced by glucose (Cu_2_O_GL).

**Figure 5:**
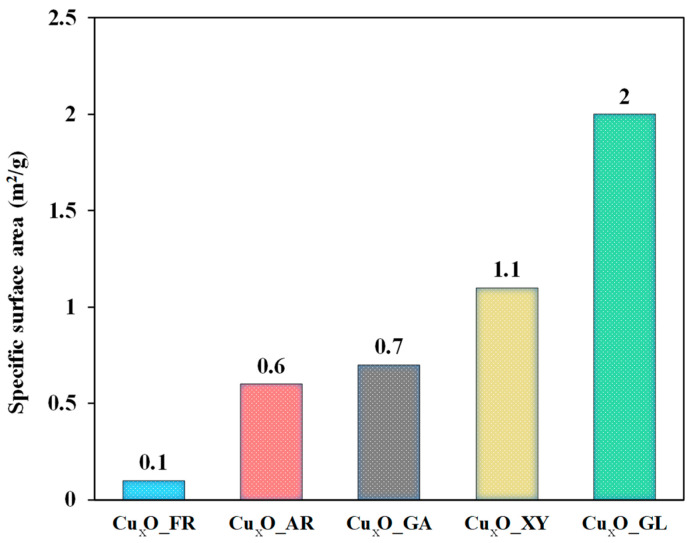
Specific surface area of the Cu_2_O samples—N_2_ adsorption measurements (BET).

As shown in Figure 5, the small specific surface area values also proved that hierarchical systems were not composed of smaller crystallites. The sample with the lowest specific surface area was Cu_2_O_FR (0.1 m^2^/g) and the largest is Cu_2_O_GL (2 m^2^/g). It is well known that the accuracy of N_2_ adsorption (Brunauer–Emmett–Teller - BET) analysis is very low in the region of the above-mentioned specific surface area values. However, there is some surprising correlation between photocatalytic activity and specific surface areas. Differences in surface area of the samples can also contribute to the observed differences in photocatalytic activity.

Regarding micrometer sized photocatalyst particles and their very low specific surface areas, aspects responsible for the enhanced photocatalytic activity should be discovered elsewhere. The possible role of interparticle voids seems to be a plausible solution, in turn, bulky microcubes in SEM images (Figure 1) do not make it very probable that they possess notable extent of porosity. Nevertheless, such a significant difference in photocatalytic activities of samples highlights the importance of using different sugars. One hypothetical reason could be an instrumentally undetectable constraint, which is caused by the diversity of sugar molecules.

## 3. Materials and Methods

### 3.1. Materials

In the syntheses, copper (II) chloride dihydrate (CuCl_2_ 2H_2_O, Alfa Aesar, Karlsruhe, Germany, 99+%) was used as precursor. As stabilization agent, ethylenediaminetetraacetic acid (EDTA, C_10_H_16_N_2_O_8_, Molar, Halásztelek, Hungary, 99.5%) was used. Additionally, sodium hydroxide—NaOH (Molar, Halásztelek, Hungary, 99.98%) was used as precipitation agent and d-(+)-glucose (Acros Organics, Morris Plains, NJ, USA, 99%), d-(+)-fructose (Alfa Aesar, Kandel, Germany, 98+%), d-(+)-xylose (Alfa Aesar, Kandel, Germany, 98+%), d-(+)-galactose (VWR, Radnor, PA, USA, 98+%) and d-(+)-arabinose (Sigma Aldrich, St Louis, MO, USA ≥99%) as reducing agents. For the purification step, Milli-Q water and acetone (VWR, ≥99.5%) were used. All the chemicals were used without further purification.

### 3.2. Characterization Methods

X-ray diffractograms (XRD) were acquired by a Rigaku Miniflex II diffractometer (Prague, Czech Republic) using Cu-Kα radiation (λ = 1.5406 Å), equipped with a graphite monochromator. Data points were taken in the 2θ° = 20–80° range at a scan speed of 1·(2θ°)∙min^−1^ [42].

Scanning electron microscopy (SEM) micrographs were recorded with a Hitachi S-4700 Type II FE-SEM (Tokyo, Japan) instrument, which operates using a cold field emission gun (5–15 kV). The size distribution of the particles was estimated from the SEM micrographs (100 particles were measured) using the ImageJ 1.52d software (Bethesda, MD, USA).

A JASCO-V650 (USA, Portland) spectrophotometer with an integration sphere (ILV- 724) was used for measuring the diffuse reflectance spectroscopy (DRS) spectra of the samples (λ = 300–800 nm). The possible electron transitions were evaluated by plotting dR/dλ vs. λ, where R is the reflectance and λ is the wavelength [43], while the indirect band gap of the photocatalysts was determined via the Kubelka–Munk method [44].

The N_2_ adsorption experiments (BET) were performed to calculate the specific surface areas of the samples, for which a BELCAT-A (Osaka, Japan)device was used to record the isotherms at 77 K.

### 3.3. Assessment of the Photocatalytic Efficiencies

A photoreactor system with 4 × 24 W visible light lamps (irradiation time = 120 min) was used to measure the photocatalytic activities. The reactor was thermostated at 25 °C using 1 M NaNO_2_, to eliminate any UV irradiation. The photocatalyst suspension containing the pollutant (initial concentration of methyl orange (MO) C_0_,_MO_ = 30 µM; catalyst content C_photocatalyst_ = 1.0 g·L^−1^; total volume of the suspension V_susp_ = 100 mL) was continuously purged with air to keep the concentration of dissolved oxygen constant during the whole experiment. The concentration decrease of the organic substrate was followed using an Agilent 8453 spectrophotometer (Agilent Technologies, Waldbronn, Germany) at 464 nm. It is important to mention that the photolysis of MO under the applied conditions was negligible [11].

## 4. Conclusions

Cu_2_O semiconductor photocatalysts were prepared under the influence of five different reducing sugars. The differences in optical properties, crystal structure, and photocatalytic activity of the samples were highlighted by examining the effect of the reducing sugars.

It seems that the particle size distribution was the most sensitive to the induced differences in the reaction mixture. The reducing agents used caused a wide size distribution spectrum of the particles from 250 nm to 2.5 μm. For each sample, usually a ~1.2 μm size was determined.

A significant difference can be observed as a function of the number of carbon atoms in the reducing sugars: using reducing agents containing five carbon atoms, a new peak appears at 722 nm in the first-order-derivative of the DRS spectra. This peak is assigned to the localized surface plasmon resonance (LSPR) of the Cu_2_O particles, which is also related to the degree of crystallinity, as this peak appeared in the spectra of the highly crystalline samples. The change in the ratio of the conduction- (at 639 nm) and the exciton bands (at 595 nm) and the change in the specific surface area of copper(I) oxide particles explains the magnitude of the photocatalytic excitability. Moreover, in spite of the low specific surface area, a very high adsorption of MO and a high photoactivity were observed, pointing out the importance of the surface quality pointed out by the DRS spectra of the samples.

## Data Availability

Not applicable.

## Sample Availability

Not available.
